# Teaching differential diagnosis in primary care using an inverted classroom approach: student satisfaction and gain in skills and knowledge

**DOI:** 10.1186/s12909-015-0346-x

**Published:** 2015-04-01

**Authors:** Stefan Bösner, Julia Pickert, Tina Stibane

**Affiliations:** 1 Department of Family Medicine, University of Marburg, Karl-von-Frisch-Strasse 4, 35043 Marburg, Germany; 2 Marburg Interactive Skills Lab (MARIS), Faculty of Medicine, University of Marburg, Marburg, Germany

**Keywords:** E-learning, Inverted classroom, General practice, Differential diagnosis, Blended learning

## Abstract

**Background:**

Differential diagnosis is a crucial skill for primary care physicians. General practice plays an increasing important role in undergraduate medical education. Via general practice, students may be presented with an overview of the whole spectrum of differential diagnosis in regard to common symptoms encountered in primary care. This project evaluated the impact of a blended learning program (using the inverted classroom approach) on student satisfaction and development of skills and knowledge.

**Methods:**

An elective seminar in differential diagnosis in primary care, which utilized an inverted classroom design, was offered to students. Evaluation followed a mixed methods design: participants completed a pre- and post-test, a questionnaire, and a focus group discussion. Interviews were transcribed verbatim and answers were grouped according to different themes. Test results were analysed using the Wilcoxon matched-pairs signed-ranks test.

**Results:**

Participants (n = 17) rated the course concept very positively. Especially the inverted classroom approach was appreciated by all students, as it allowed for more time during the seminar to concentrate on interactive and practice based learning. Students (n = 16) showed a post-test significant overall gain in skills and knowledge of 33%.

**Conclusions:**

This study showed a positive effect of the inverted classroom approach on students’ satisfaction and skills and knowledge. Further research is necessary in order to explore the potentials of this approach, especially the impact on development of clinical skills.

**Electronic supplementary material:**

The online version of this article (doi:10.1186/s12909-015-0346-x) contains supplementary material, which is available to authorized users.

## Background

One of the essential skills of the clinician is the ability to make an accurate diagnosis. Especially in the field of primary care the process of making a differential diagnosis is a challenging and sometimes daunting task. Diseases often present at an early stage and sometimes in an atypical form, therefore primary care physicians use a broad range of diagnostic strategies [1]. Medical students are usually taught differential diagnosis in the high prevalence setting of a university hospital. In addition, differential diagnosis of disease symptoms with a broad underlying aetiology (e.g. dizziness) will be split among different disciplines (such as neurology, ENT and internal medicine). However, many students will later work as physicians in the low prevalence context of primary care, where they will have to deal with the diagnostic uncertainty that is connected with seeing patients with a broad range of symptoms [2]. General practice/family medicine, which plays an increasingly important role in undergraduate medical education throughout Europe [3], is in an excellent position to give students an overview of the whole spectrum of differential diagnosis in regard to common symptoms encountered in primary care.

Nowadays, e-learning is an integral part of medical education, and has been shown to be most effective when combined with face-to-face teaching, facilitating the so-called blended learning approach [4-6]. Students increasingly use mobile devices like smartphones for learning purposes, and appreciate being able to utilise multimedia materials like video clips [7] or podcasts [8] for the preparation or revision of course content. Undergraduate medical students highly value the use of e-learning, especially when integrated into a blended approach [9]. While blended learning approaches are well established in the postgraduate education of primary care providers [10,11], there are fewer examples in the field of undergraduate medical education in primary care [12].

The ‘inverted’ or ‘flipped classroom’ approach takes blended learning one step further, as it offers all traditional lectures exclusively via e-learning, and uses the face-to-face sessions for interactive exercises and critical reflection [13]. This concept is already widely used in other disciplines, for example in the social sciences. Different theoretical foundations to justify the inverted classroom approach derive from the literature on student-centered learning including Piaget’s theory of cognitive conflict and Kolb’s theory of experiential learning [14]. It has been shown that this approach positively influences students’ openness for cooperative learning and innovative teaching methods [15]. While students still preferred live lectures to video lectures, they valued the interactive class time higher than in-person lectures [14].

Inverted classroom education has just started to be incorporated into medical education [16], and there are so far only few published examples [17-20], however none of these in the field of primary care. In contrast, we consider especially the field of primary care learning as highly suitable for an inverted classroom approach. Primary care includes a broad range of complaints and diseases that can be well taught via e-learning. Class time can then be effectively used to teach the complex process of medical decision making in patients with a broad range of symptoms, the typical setting of primary care, and to explore ways how to deal with the connected uncertainty.

We redesigned a seminar on differential diagnosis in primary care for undergraduate students, using an inverted classroom approach. In this study we wished to address the following questions:

1. Will students appreciate this approach for studying differential diagnosis in primary care?
2. What will the gain in skills and knowledge of students learning with the inverted classroom approach be?

## Methods

### Participants and setting

All participants of this elective seminar were in their fourth or fifth year of undergraduate medical studies. The different modules consist of interdisciplinary clinical pictures derived from the everyday routine of primary care. Contrary to other clinical specialties, where students are also presented with very rare diseases which they may rarely -if ever- encounter during their medical career, we primarily concentrated on common underlying aetiologies for lead symptoms such as chest pain, dyspnoea, abdominal pain, or vertigo/dizziness. A special focus was put on the diagnostic accuracy of symptoms and signs in regard to the different underlying disease aetiologies of a given clinical picture. The seminar comprises of 42 hours of face-to-face teaching altogether, divided over 3 hours per week respectively, and closes with an objective structured clinical examination (OSCE). The whole seminar takes place at an interactive skills lab attached to the Marburg University Hospital and utilizes trained simulation patients and different models. Further details of seminar content and underlying didactic considerations have been published elsewhere [21].

One year ago, we redesigned the whole seminar, using the inverted classroom approach [22,23]. At the faculty of medicine, our seminar is currently the only taught medical course using the inverted classroom design. To our knowledge, this method is so far also not used by other primary care departments in Germany and in general not yet widely established in medical education at German Universities.

E-learning modules are hosted on the web based learning platform ‘K-Med’ (knowledge in medical education) of the faculty of medicine, University of Marburg. Each seminar session is structured as follows:

- Preparation: Several video and audio lectures giving introductory information and key knowledge content are available on the web-based learning platform.
- Face-to-face teaching, which takes place in the interactive skills lab: several didactic approaches, such as simulation patients, training models, interactive small group work, and quiz exercises are used.
- Follow-up: Additional video and audio lectures present more detailed information in regard to the single leading symptom and its underlying etiologies. Supplementary facultative reading material (e.g. original research on the accuracy of symptoms and signs for a given disease) is also offered via the web-based learning platform.

### Study design

We used a mixed methods design in order to investigate our study questions.

Student satisfaction (research question 1) was measured using information from different sources. The first source was a standardized questionnaire that is used by the University of Marburg to evaluate seminars. The questionnaire utilizes a rating scale ranging from 1 point (“agree not at all”) to 5 points (“completely agree”) and additional free text answers, and covers four main areas (seminar concept and presentation, interaction with students, level of interest/relevance and difficulty/quantity/speed). It is filled out anonymously and analysed by the university’s evaluation department.

In addition, after the 8th out of 14 course sessions, an evaluation consisting of a focus group discussion and a short questionnaire that was handed out after the focus group were conducted. Topics of the interview and the questionnaire included the perception of the seminar content, a critical reflection of the inverted classroom approach (usefulness of video and audio sessions, connection/synthesis with the face to face sessions) and the individual learning experience of the students. We concentrated on the learning experience of the course participants, with a special focus on how the inverted classroom approach is perceived. For the quantitative evaluation of gain in skills and knowledge (research question 2) we designed a questionnaire consisting of extended matching items (13 items), and key-feature tests (20 items). Both examination formats can measure the process of clinical reasoning, and help to assess clinical decision making skills [24,25]. They are used to test the second level of clinical competence in “Miller’s Pyramid” (“Knows how”) [26]. We found the extended matching questions suitable for testing which clinical findings from the patient’s history and physical examination had the highest diagnostic accuracy.

Together, both question formats covered all major seminar content. Questions were pre-tested on another group of students with comparable pre-existing knowledge. After the pre-test we replaced two key feature cases that were too easy and one extended matching question that was too difficult and in addition modified one key feature question that was misleading. During the seminar, students were asked to complete the pre-test at the beginning of the first session, and the post-test after the last session before starting to learn for the OSCE that was conducted one week later. Tests were completed by students using an unique identifier that allowed the anonymous matching of pre- and post-tests at an individual student level.

Additional file 1 shows example questions of the questionnaire.

### Analysis

For the 4 variables of the standardized questionnaire for student satisfaction means and standard deviations were calculated and plotted.

Results (average in percentage of maximal test score that could be gained) of the pre- and post-test were compared for statistically significant differences using the Wilcoxon matched-pairs signed-ranks test. Error probability with a p-value less than 0.05 was considered significant. Statistical analysis was performed using the GraphPad PRISM (Version 6 Graphpad Software, Inc).

The focus group discussion was taped and transcribed verbatim. For data analysis we utilised a deductive approach based on the questions of the focus group guideline and of the short written questionnaire. Analysis was performed by SB and results discussed among all authors. Responses from the focus groups and the free text answers from the evaluation questionnaires were grouped under different themes.

The entire here presented data are part of the routine evaluation of courses at our faculty. Ethical approval was therefore not required.

## Results

Altogether, 17 students applied and were all enrolled in the seminar. One student did not participate in the pre-test. All 17 students took part in the focus group and the final evaluation.

### Student satisfaction

**Figure 1:**
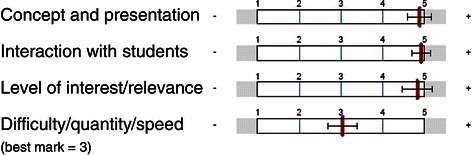
**Global results of the summative evaluation (n = 17).** 5 points are the best possible result; the category ‘difficulty/quantity/speed’ has 3 points as the best possible result. Red bars reflect mean values and horizontal bars reflect standard deviations.

Figure 1 shows the summary of results concerning the four areas investigated in the quantitative evaluation. The inverted classroom concept of the seminar reached the highest possible marks. Interaction with students and the relevance of the course contents were rated accordingly. The quantity of material offered, and the complexity of course content were rated as completely adequate.

Answers from the focus group discussion and the evaluation questionnaires can be grouped under the following themes:

#### Seminar content

All students appreciated the symptom-oriented approach, which was seen to reflect the reality of daily practice.

*“Usually you learn from a disease-oriented point of view…but the patient usually presents with a symptom. This is what usually lacks* (in other seminars or lectures)*… it is difficult to remember all the differential diagnoses, because you have to screen all diseases that you know in order to see whether these contain the symptom… here* (in this seminar) *it is the other way around.”.*

Most students mentioned that the seminar helped to understand the importance of epidemiological knowledge for the diagnostic process and the integration of this knowledge into the process of clinical decision making.

*“Epidemiology was a topic which I considered as totally uninteresting, but now its importance has become clearer to me.”*

*“There is a much more frequent reference to epidemiology* (in comparison to other seminars/lectures), *which I consider good, as it helps to build a much stronger hierarchy of differential diagnoses.”*

#### Inverted classroom approach

The blended learning design of the seminar using an inverted classroom approach was appreciated by all students, as it gave more time during the seminar to concentrate on interactive and practice based learning.

*“The things* (seminar content) *that are in the videos can be outsourced very easily and there is no necessity to cover them here* (during the face to face lessons)*.”*

*“The summaries of different diseases in the preparatory lessons are very good. The immediate application with the help of simulation patients is very practical.”*

Some students criticized the fact that key information of the preparation videos was repeated during the face-to-face sessions.

*“One can be even more radical; we have already seen the video and you then don’t need the slides anymore.”*

#### Acquisition of further key competencies

Several students mentioned the positive impact of teamwork during the seminar – a key competence that was also considered as important for later professional life.

*“It is very good that we have the opportunity to reason together as a team…to brainstorm together in order to consolidate what we have heard.”*

*“It is also good to get ideas from others. I think that this is also important for being a doctor later, to learn this capacity for teamwork.”*

For some students the seminar was also a trigger for meta-learning, as they reflected their own learning experience.

*“I learn best when I need to apply my previous knowledge to unknown new situations that I consider difficult. Then I am most creative and it* (the knowledge) *remains in the long-term memory.”*

### Gain in skills and knowledge

**Figure 2:**
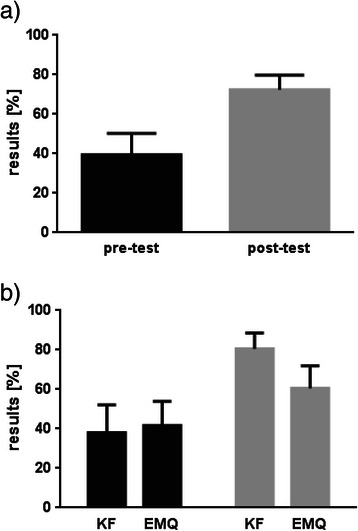
**Overall absolute gain in skills and knowledge and test results stratified according to test format (n = 16).** KF = Key feature questions, EMQ = Extended Matching Questions. Upper part: Overall results of the written pre- and post-test including confidence intervals. Percentages of the total number of possible points are presented indicating absolute gain in skills and knowledge. Students showed significant improvement (p < 0.01). Lower part: Results of the written pre- (black color) and post-test (grey color) stratified by test format. For both formats a significant improvement could be demonstrated (p < 0.01).

There was a significant (p < 0.01) overall absolute gain in skills and knowledge of 33%. Gain of fourth year students was slightly higher at 34% than that of fifth year students (31%). Students showed a higher gain at 42% in the key feature questions compared to extended matching questions (19% gain). Figure 2 shows overall results and results stratified by test format.

**Figure 3:**
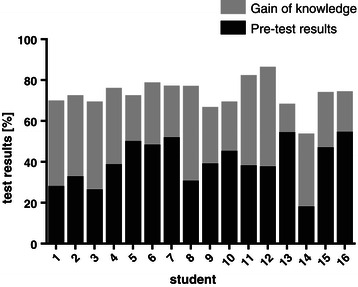
**Individual gain of participating students (n = 16).** Gain in skills and knowledge of each individual course participant.

Figure 3 presents results of individual students that showed a large heterogeneity and ranged between 13-48%.

## Discussion

We aimed to give a first account of the effects of a blended learning program in primary care with this study. To our best knowledge the above presented seminar is the first to teach differential diagnosis in primary care for undergraduate students using an inverted classroom approach. Our evaluation showed both a high satisfaction rate and a significant gain in skills and knowledge.

When planning to introduce a new e-learning program it is crucial to evaluate participants’ reception of the chosen teaching approach [5,27]. The high approval of our blended learning approach is consistent with the available literature. While most studies show a positive effect of blended learning on students’ satisfaction [5,8,28], not all students necessarily appreciate and use multimedia materials such as video clips [7].

A recently conducted Delphi study on the development of a technology-mediated teaching strategy, the authors called for teaching activities that are “learner-centred, interactive, integrated, reflective and that promote engagement” [29]; the inverted classroom approach was mentioned among others as one possible strategy to achieve this goal. Our finding of high student satisfaction with this approach especially, is supported by the – presently sparse- literature in the field of medical education. While we could not identify evaluations of other primary care programs that use the inverted classroom model, there have been programs in teaching palliative care skills [19], cardiovascular, respiratory and renal physiology [20], renal pharmacotherapy [17] and perfusion skills [18]. All of the above mentioned programs used the inverted classroom approach and were positively evaluated, which supports our own findings.

An additional finding in our focus group discussion was that the new learning experience during the seminar also triggered part of the participants to critically reflect on their own learning. Biggs refers to this process as ‘metacognition’ which includes the awareness of the own cognitive processes together with exerting control over them. He encourages a deep approach to learning which includes the promotion of guided self-questioning, using other students as a resource or to derive own heuristics to suit a given task [30]. All these mentioned elements can be found in our course and it is interesting to note that an innovative and unusual teaching approach can trigger this kind of critical self-reflection, which we consider an important prerequisite for later professional life.

While a broad acceptance of participating students is surely a precondition for establishing a successful blended learning program [31], there should also be a positive impact on clinical skills. In one study students rated e-learning just as highly as alternative traditional methods of clinical skills teaching [9]. However, the effectiveness of blended learning is often difficult to quantify [5,6]. We observed a gain in skills and knowledge of the participating students in the course of our seminar, but cannot judge what additional effect the blended learning/inverted classroom approach had in comparison to usual face-to-face teaching. In addition, the results of a systematic review on the role of blended learning in the clinical education of health-care students, which identified only a few high quality studies on this topic, provided only rudimentary evidence that technology-enhanced teaching improves clinical competencies [32].

There are strengths, but also several limitations to our study. The chosen mixed methods study design is an appropriate way to evaluate a blended learning program, taking qualitative and quantitative information into account [33]. We not only evaluated student satisfaction, but also tried to quantify gain in skills and knowledge at both a group and at an individual level. One limitation is the relatively small number of participants included in the study. As the seminar is highly interactive, consisting mainly of work in small groups with tutors and simulation patients, no more than 18 students can be enrolled. This is also one of the reasons why we could not compare the inverted classroom approach with a second group taught using traditional methods. This would have been desirable from a methodological point of view; however it was not feasible to divide the small group of participants any further. As we do not have long term follow up results (e.g. repetition of the post-test after 6 months), we cannot say how much knowledge was retained by the students. Both examination formats that we used can only test the second level of clinical competence according to “Miller’s Pyramid”. It would have been desirable to perform an OSCE as pre- and post-test. However, from a practical point of view we did not consider it appropriate to confront students with the complex challenges of an OSCE at the start of the seminar. In this instance we had to balance methodological considerations versus the danger of frustrating the participating students right at the beginning of this elective course with an OSCE they would not at all be prepared for.

## Conclusions

This study shows that an inverted classroom model is well suited to teach the complex topic of differential diagnosis in primary care. While we perceived high student satisfaction, we could not prove whether the inverted classroom approach leads to a higher gain in skills and knowledge than traditional face-to-face teaching. Future research on our presented concept should therefore aim for a direct comparison of these two teaching approaches on gain in skills and knowledge, ideally in the form of a randomized controlled trial with an OSCE as test format.

## Additional file

Additional file 1:
**Example questions.**
