# Supplementary material for: Teaching differential diagnosis in primary care using an inverted classroom approach: student satisfaction and gain in skills and knowledge
Source: BMC Med Educ. 2015 Apr 1;15:63. doi: 10.1186/s12909-015-0346-x (PMC4404043; doi:10.1186/s12909-015-0346-x)
Supplement: Additional file 1: — Example questions. [file 12909_2015_346_MOESM1_ESM.docx]

Additional file 1: Example questions

Example key feature question:

Acute Vertigo

Ms. Schneider, a 52 year old patient, presents in your GP practice. She reports that she had repeated episodes of acute vertigo while still lying in bed. Each episode lasted about 1 minute. When she was changing her position vertigo started again. Right now she does not feel dizzy but she is very concerned that something serious might have happened to her.

**Quest ion 1: What is your suspected diagnosis?**

**………………………………………………………………………………………………………………………………………………………**

**Question 2: Which examination is most likely to confirm your tentative diagnosis?**(Please circle the most suitable answer)

1) You ask the patient to stand in upright position. After some minutes you ask the patient to close her eyes.

2) You position yourself in front of the patient and take her head between your both hands. You ask her to fixate a certain point in the room with her eyes. You rotate the patient’s head with a quick movement.

3) You ask the patient to walk with her eyes closed on a straight line. Like a tight rope walker she shall slowly set one foot before the other.

4) You perform an otoscopy. Typical changes of the tympanic membrane will lead to the diagnosis.

5) You ask the patient to sit down on your examination couch. You take her head and turn it 45° to one side. Then you quickly change the position of the patient from supine to lying so that her head is reaching slightly beyond the upper end of the examination couch.

6) You first measure the blood pressure of the patient in supine, then in lying position. You ask the patient to remain in a lying position for about 3 minutes and then to quickly stand up. You measure the blood pressure again.

7) You ask the patient to lie down on the examination couch and you alternately apply first warm (37°C) and then cold water into both ears.

8) You perform a hearing test for both ears and check for air and bone conduction.

9) None of the above.

**Question 3: What is the next step that you undertake?**(Please circle the most suitable answer)

1) You refer your patient to the radiology department; as symptoms developed rapidly, a malignant process should be excluded and a cranial MRI scan should be performed.

2) You prescribe Metoclopramide in liquid form which the patient should take in case of nausea or vomiting.

3) As the ear cartilage seems to be tender and any bacterial infection could spread locally you prescribe Amoxicillin for 7 days.

4) In order to prevent damage of the inner ear you prescribe Azithromycin for 3 days and add Dimenhydrinat (Vomex) against the nausea.

5) You demonstrate some positioning exercises to the patient that she can do herself should symptoms reappear.

6) You give the patient an initial dose of steroids and refer her to the local ENT department for further treatment.

7) You explain your patient that dizziness and vertigo have often underlying psychological problems and you offer to meet again in order to talk about possible underlying stressful life events.

8) In order to speed up the healing process you prescribe antibiotic ear drops. In addition the patient shall apply nasal spray for at least one week.

9) You refer the patient to the cardiologist who shall check the vascular status of the patient (including an ultrasound of the carotid arteries) and perform a 24 hour blood pressure measurement.

10) You take blood and order further tests (blood picture and inflammatory markers) and prescribe Amoxicillin for 7 days.

11) None of the above.

Example extended matching question:

Which two items of the patient’s history and/or physical examination are most helpful for the General Practitioner in relation the respective suspected diagnosis?

Choose from the list and write the 2 answers (numbers) behind each case. Answers can apply for more than one case description.

Case 1:

Ms Maurer, a 37 year old patient, has made an appointment as she is suffering already for a longer time from episodic dyspnea sometimes linked to physical exercise. As a child she was suffering from neurodermitis which has resolved during puberty. Your suspected diagnosis is asthma.

Case 2:

Mr Passauer, a 62 year old patient has been treated during the last year for multiple respiratory tract infections. You know that he has been smoking for many years. Your suspected diagnosis is chronic obstructive pulmonary disease.

Case 3:

You are called by Ms Kahl for a home visit. The 86 year old patient has dyspnea since this morning which has been increasing, especially when lying down. You know the patient. Your suspected diagnosis is acute heart failure.

Case 4:

Ms Meier (23 years) visits your practice; she has arrived this morning by plane from Australia and is suffering now from chest pain depending on breathing. Your suspected diagnosis is pulmonary embolism.

Case 5:

Mr Kautz (52J.) visits your practice. He has high temperature, feels unwell. You listen to his lungs. Your suspected diagnosis is pneumonia.

1) Dyspnea with wheezing

2) Hemoptysis

3) Symptoms of angina during night hours

4) Basal rhonchi

5) Age above 45 years

6) Increased production of sputum

7) Chest pain

8) Diarrhea

9) Deep vein thrombosis or pulmonary embolism in the past history

10) Decreased breathing sound

11) Nocturnal dyspnea

12) Dyspnea

13) Oedema of both lower legs

14) Third heart sound

15) Smoking history of > 70 pack years

16) Raised jugular venous pressure

17) Any heart murmur

18) The patient reports suffering from chronic obstructive pulmonary disease
